# Optimization and Analysis of Tangential Component Orientations in OPM-MEG Sensor Array

**DOI:** 10.3390/bioengineering12090903

**Published:** 2025-08-22

**Authors:** Wenli Wang, Fuzhi Cao, Nan An, Wen Li, Chunhui Wang, Zhenfeng Gao, Min Xiang, Xiaolin Ning

**Affiliations:** 1 Key Laboratory of Ultra-Weak Magnetic Field Measurement Technology, Ministry of Education, School of Instrumentation and Optoelectronic Engineering, Beihang University, Beijing 100191, China; zb1917006@buaa.edu.cn (W.W.); caofuzhi@buaa.edu.cn (F.C.); chunhuiwang@buaa.edu.cn (C.W.); gaozhenfeng@buaa.edu.cn (Z.G.); xiang_min@buaa.edu.cn (M.X.); ningxiaolin@buaa.edu.cn (X.N.); 2 Hangzhou Institute of National Extremely-Weak Magnetic Field Infrastructure, 465 Binan Rd., Binjiang District, Hangzhou 310051, China; 3 School of Engineering Medicine, Beihang University, Beijing 100191, China; 4 Hefei National Laboratory, 96 Jinzhai Rd., Gaoxin District, Hefei 230088, China; 5 Zhejiang Provincial Key Laboratory of Ultra-Weak Magnetic-Field Space and Applied Technology, Hangzhou Innovation Institute, Beihang University, Hangzhou 310051, China

**Keywords:** magnetoencephalography, OPM-MEG, tangential component, array sensitivity, lead field correlation coefficients, source localization

## Abstract

Optically pumped magnetometers (OPMs) have brought a transformative advancement to magnetoencephalography (MEG), enabling flexible, noncryogenic, and wearable neuroimaging systems. In particular, the development of triaxial OPM sensors allows for simultaneous measurement of full magnetic field vectors, including both radial and additional tangential components. Previous studies have shown that incorporating tangential components helps enhance the separation between neural signals and external interference, but their optimal configurations remain unclear. This study systematically investigated the impact of tangential component configurations on array sensitivity and the lead field correlation coefficient ($R_{12}$) in triaxial OPM-MEG sensor arrays, considering tangential component rotations, relative orientations of sensor and source, source depths, and head model types. Based on the above analysis, we proposed an optimization strategy aimed at minimizing $R_{12}$, referred to as $R_{12}$-minimization array optimization (RMAO), to explore the optimal configuration of tangential components. The simulation results showed that the proposed method significantly enhanced sensitivity to cortical sources and effectively suppressed external interference, enabling more accurate source localization. This study highlights the critical role of tangential components in improving system performance and provides theoretical foundation and methodological guidance for the design of triaxial OPM-MEG sensor arrays.

## 1. Introduction

Magnetoencephalography (MEG) is a noninvasive neuroimaging technique that indirectly infers brain activities by measuring the magnetic fields derived from neural currents [1,2]. MEG offers exceptional temporal resolution along with good spatial resolution, making it a valuable tool in both neuroscience research and clinical applications [3,4,5,6]. Traditional MEG systems rely on superconducting quantum interference devices (SQUIDs), which, despite their high sensitivity, require cryogenic cooling and are rigidly housed in dewar containers [7,8]. This design constrains the sensor-to-scalp distance to approximately 2 cm, thereby limiting signal strength.

The advent of optically pumped magnetometers (OPMs) has marked a significant advancement in MEG technology [9,10]. As a new generation of quantum sensors, OPMs operate at room temperature and can be placed directly on the scalp, enabling substantially higher sensitivity to cortical sources [11,12]. In addition, the latest generation of triaxial OPM sensors is capable of measuring not only the radial component perpendicular to the scalp surface but also two tangential components [13]. Prior studies have shown that incorporating tangential components enriches spatial information, significantly improves source localization accuracy, and enhances the reliability of source orientation estimates [14,15]. Moreover, this multidirectional sensing capability greatly improves interference suppression, particularly when applying spatial filtering methods such as Signal Space Separation [16]. The tangential sensitivity of the OPM array also makes it excellent for detecting deep brain sources, such as those in the hippocampus, by yielding stronger signal amplitudes and improved localization accuracy [17]. Additionally, the inclusion of tangential components enables more uniform cortical coverage and maintains high sensitivity even when the number of recording channels is limited, making OPM-MEG systems more suitable for wearable and motion-tolerant neuroimaging applications [18].

Despite these advantages, the optimal configuration strategy for tangential components in the triaxial OPM-MEG system remains insufficiently studied. In particular, the impact of the orientation and spatial distribution of tangential components on neural source sensitivity and interference suppression has yet to be systematically investigated. To address this gap, this study systematically evaluated the impact of factors such as tangential component rotation, relative orientation of sensor and source, source depth, and head model type on the performance of triaxial OPM-MEG systems through simulation experiments, with a focus on key metrics including array sensitivity and lead field correlation ($R_{12}$). On this basis, an optimization strategy was proposed, referred to as $R_{12}$-minimization array optimization (RMAO), which aims to identify the optimal configuration of tangential components. The simulation results demonstrated that this strategy effectively enhances neural source sensitivity and localization accuracy.

## 2. Materials and Methods

### 2.1. Triaxial OPM Sensor

**Figure 1:**
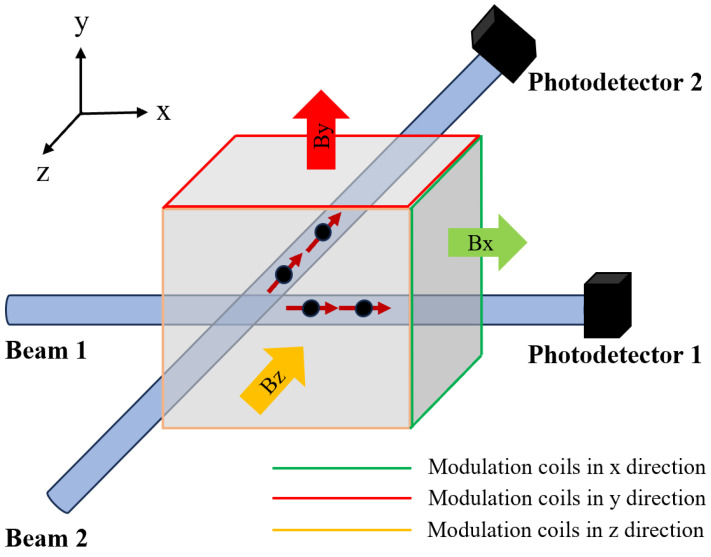
Schematic of the triaxial OPM sensor.

The triaxial OPM sensor (QZFM Gen-3, QuSpin Inc., Louisville, CO, USA) primarily consists of a laser, a glass vapor cell, two sets of independent photodetectors, and three mutually orthogonal pairs of electromagnetic coils (as illustrated in Figure 1). The laser emits light at a specific frequency or wavelength, which is split into two beams by a beam splitter. These beams are directed into the vapor cell along orthogonal directions. After traversing the vapor cell, the intensity of each beam is detected by a corresponding photodetector and converted into an electrical signal. Specifically, the first beam is sensitive to magnetic fields along the x and y axes, while the second beam is sensitive to fields along the y and z axes. By combining the measurements from both beams, the full vector magnetic field can be reconstructed. To ensure accurate measurement, the vapor cell is surrounded by three orthogonal electromagnetic coils, which maintain a near-zero magnetic field environment and provide oscillating modulation fields to facilitate lock-in amplifier-based signal processing.

### 2.2. Measurement Model

To analyze the differences in source localization accuracy and interference immunity among various sensor array designs, we assume that the MEG data includes an intracerebral source of interest and an extracerebral source of interference. Under this assumption, the measured MEG data can be represented as

(1) $$Y=L_{1}Q_{1}+L_{2}Q_{2}+E$$

where Y represents the magnetic field amplitudes at n sensor channels at time t; $L_{1}$ is the forward field for the source of interest $Q_{1}$ in the brain; $L_{2}$ is the forward field for the source of interference $Q_{2}$ out the brain; and E is the additive noise.

The location of the interested source $Q_{1}$ in the brain can be estimated using

(2) $$\hat{Q_{1}}=W_{1}(L_{1}Q_{1}+L_{2}Q_{2}+E)$$

where $\hat{Q_{1}}$ is the estimated source; $W_{1}={\left(L_{1}^{T}C^{-1}L_{1}\right)}^{-1}L_{1}^{T}C^{-1}$, which is the beamformer spatial filter [19]. C is the data covariance.

To quantify the total error across an entire time course, $E_{tot}^{2}$, we use the sum of the squared differences between the reconstructed and original time courses as an evaluation metric [15]

(3) $$E_{tot}^{2}=\sqrt{\sum_{i=1}^{M}{\left({\hat{Q}}_{1i}-Q_{1i}\right)}^{2}}$$

where M  is the total number of time points during the recording and i denotes a single time point.

In the simplest case, $E_{tot}^{2}$ can be expressed as the sum of the interference source error, $E_{Q_{2}}^{2}$, and the sensor noise error, $E_{noise}^{2}$, when the sensor noise and both sources are independent of each other [15]

(4) $$E_{tot}^{2}=E_{Q_{2}}^{2}+E_{noise}^{2}=\frac{Q_{2}^{2}{\left\|L_{2}\right\|}^{2}}{{\left\|L_{1}\right\|}^{2}}R_{12}^{2}{\left[\frac{1-f_{2}}{1-f_{2}R_{12}^{2}}\right]}^{2}+\frac{v^{2}}{{\left\|L_{1}\right\|}^{2}}\left(\frac{1+f_{2}^{2}R_{12}^{2}-2f_{2}R_{12}^{2}}{\left(1-f_{2}R_{12}^{2}\right)}\right)$$

where $\left\|L_{1}\right\|$ and $\left\|L_{2}\right\|$ are the Frobenius norm of the lead field matrix $L_{1}$ and $L_{2},$ respectively. They are measures of how the sensor array is affected by the source, referred to as the array sensitivity. $R_{12}=\;\frac{L_{1}^{T}L_{2}}{\left\|L_{1}\right\|\left\|L_{2}\right\|}$; it is a measure of the similarity of the lead field patterns for sources $Q_{1}$ and $Q_{2}$, referred to as the lead field correlation coefficient. $f_{2}=\;\frac{Q_{2}^{2}{\left\|L_{2}\right\|}^{2}}{v^{2}+Q_{2}^{2}{\left\|L_{2}\right\|}^{2}}$; it represents a scaled signal-to-sensor noise ratio. v  is the standard deviation of the noise at each sensor, which we assume is equal across sensors and is an inherent property.

It has been shown in previous studies that the influence of the parameter $\left\|L_{1}\right\|$ depends on the number of sensors, and the larger the $\left\|L_{1}\right\|$ is, the smaller the total beamformer error is. The parameter $R_{12}$ depends closely on the tangential components of the sensors, and the smaller the $R_{12}$ is, the more separable the source is, which means the better the immunity to interference [15]. Therefore, the parameters $R_{12}$ and $\left\|L_{1}\right\|$ are the important factors affecting the total error of the beamformer, and the performance of the system can be optimized by appropriately designing the sensor array.

### 2.3. Effects of Tangential Components Configuration

To systematically evaluate the effects of tangential components configuration, this subsection describes the simulation scenarios and the used anatomical model, sensor array, and noise model. The simulations were performed under different tangential component rotations, relative orientations of sensor and source, source depths, and head model types.

#### 2.3.1. Anatomical Model

To simulate source activities within the brain, we collected T1 MRI images of a 27-year-old healthy female participant. The MRI data were segmented using the FreeSurfer software (Version 6) [20] and reconstructed using the Brainstorm software (Version 3.24) [21] to obtain cortical and scalp surface meshes. The cortical surface mesh was downsampled to 15,002 vertices to be used as a distributed source model. Each vertex represented a source location, and all the sources were oriented perpendicular to the local cortical surface [22].

#### 2.3.2. Sensor Array

**Figure 2:**
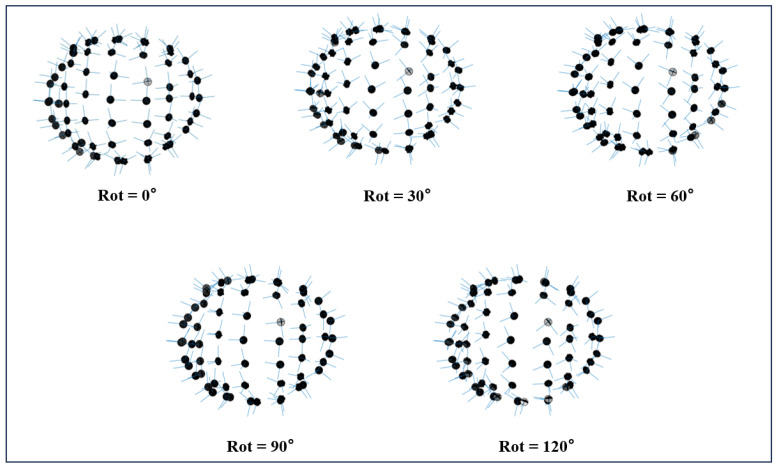
Triaxial OPM sensor arrays with different rotations.

To investigate the effect of tangential component rotation on the triaxial OPM-MEG measurement system, we first determined the sensor positions of the OPM array based on the layout of the 64-channel EEG system (BioSemi B.V., Amsterdam, the Netherlands). The radial, azimuthal, and polar directions were defined as the initial measurement axes of the sensor array (ROT = 0°). Subsequently, the tangential components of the initial sensor array were rotated clockwise around their radial axes in the plane defined by the two tangential components, with a rotational step of 30 degrees. As a result, five triaxial OPM sensor arrays with different tangential component orientations were generated (Figure 2).

#### 2.3.3. Noise Model

To evaluate the effect of tangential components on the capability of interference suppression, we constructed noise models that simulate external interference sources. The MRI data were segmented using the SPM software (Version 25.01) [23] to obtain scalp, skull, and brain surface meshes. Based on the brain surface mesh, we employed the least-squares fitting approach [24] to approximate a fitted sphere that envelopes the outer surface of the brain. The estimated center and radius of the fitted sphere were used to define a reference coordinate system for external interference sources. For each vertex on the brain surface, we identified the corresponding point on the fitted sphere along the radial direction and projected it outward by approximately ten times to simulate the interference sources located at further distances outside the brain. The orientation of each interference source was defined as the tangential orientation of the sphere.

#### 2.3.4. Evaluation Metrics

The array sensitivities of the sensor to internal and external sources ($\left\|L_{1}\right\|$ and $\left\|L_{2}\right\|$), along with the lead field correlation coefficient ($R_{12}$), were used as sensor-level evaluation metrics. The dipole localization error (DLE), defined as the Euclidean distance between the reconstructed and simulated dipole positions, was used as the source-level evaluation metric [25,26]. These metrics were employed to systematically assess the influence of tangential components on source sensitivity, interference suppression, and source reconstruction accuracy of the beamformer.

#### 2.3.5. Simulation Scenarios

(1) Explore the effects of tangential orientations

To analyze the effects of tangential component rotation on $\left\|L_{1}\right\|$ and $R_{12}$, we calculated the mean values of $\left\|L_{1}\right\|$ and $R_{12}$ for the five sensor arrays with different rotation angles constructed in Section 2.3.2. To further investigate the physical mechanism underlying the variation in $\left\|L_{1}\right\|$, we decomposed the total magnetic field sensitivity ($L_{tot}$, equivalent to $L_{1}$) into the magnetic field sensitivity generated by primary currents ($L_{prim}$, computed based on the Biot–Savart law) and that generated by volume currents ($L_{vol}=L_{tot}-L_{prim}$), and we calculated the Frobenius norm ($\left\|L_{prim}\right\|$ and $\left\|L_{vol}\right\|$) of each component. Subsequently, using the array sensitivity at ROT = 0° as the reference, we computed the array sensitivity differences ($\left\|\Delta L_{tot}\right\|$, $\left\|{\Delta L}_{prim}\right\|$, and $\left\|{\Delta L}_{vol}\right\|$) for each magnetic field component at the remaining rotation angles, and further evaluated the spatial consistency of these variations using Pearson correlation coefficients.

(2) Explore the effect of relative orientations between sensor and source

To analyze the effect of relative orientations between sensor and source, referred to as sensor–source alignment, on $\left\|L_{1}\right\|$ and $R_{12}$, we computed the cosine similarity between each source vector and its corresponding sensor measurement direction as the alignment. An alignment value of 1 indicated complete parallelism, while a value of 0 indicated orthogonality between the two vectors. Based on this, we used the ROT = 0° array as a reference and calculated the sensitivity differences ($\left\|\Delta L_{1}\right\|$) and correlation coefficient difference ($\Delta R_{12}$) at rotation angles of 30°, 60°, 90°, and 120°, respectively. The alignment values between each source orientation and the three measurement components (radial, tangential 1, and tangential 2) were grouped into five bins (Bin1–Bin5) to analyze how different levels of alignment modulated the perturbations in sensitivity and lead field structure. Pearson correlation coefficients were used to quantitatively evaluate these effects.

(3) Explore the effect of source depth

To evaluate the effects of source depth on $\left\|L_{1}\right\|$ and $R_{12}$, we calculated the shortest distance from each source point to the scalp surface and used it as the source depth metric. Subsequently, for all five sensor array rotation angles, we computed the $\left\|\Delta L_{1}\right\|$ and the $\Delta R_{12}$ at each source point and conducted Pearson correlation analyses with respect to source depth. In addition, to further investigate the overall relationship between source depth and system stability, we calculated the average $\left\|\Delta L_{1}\right\|$ and $\Delta R_{12}$ across all the rotation angles for each source point and performed a joint regression analysis.

(4) Explore the effect of head model type

To evaluate the effects of head model type on $\left\|L_{1}\right\|$ and $R_{12}$, we constructed five head models based on previous research work [27]. These include the Single Sphere model (SSP) [28], the Local Sphere model (LSP) [29], the Single Shell model (SSH) [30], the three-compartment boundary element method (BEM) [31] model, and the five-compartment finite element method (FEM) [32] model. For each head model, the lead field matrix $L_{1}$ corresponding to five sensor array configurations with different rotation angles was computed using different toolboxes. Specifically, the leading fields for SSP, LSP, and SSH were computed using the Fieldtrip toolbox (Version 20220202) [33], and the leading fields for BEM and FEM were computed using the OpenMEEG (Version 2.4) [34] and Duneuro (Version 230223) [35] toolboxes, respectively. Subsequently, we calculated the corresponding array sensitivity ($\left\|L_{1}\right\|$) and lead field correlation coefficient ($R_{12}$) for each model, and further analyzed their variations ($\left\|\Delta L_{1}\right\|$ and $\Delta R_{12}$) across different rotation angles to evaluate the modulatory effects of head models on system performance stability.

### 2.4. Array Optimization

To optimize the configuration of tangential components in the triaxial OPM sensor array, we adopted an optimization strategy based on the minimization of the lead field correlation coefficient $R_{12}$, referred to as the $R_{12}$ minimization array optimization (RMAO) method.

Assuming there are N triaxial sensors in total (with N=64 in this study), the tangential orientations of each sensor are optimized sequentially. For each sensor k∈{1,2,…,N}, its two orthogonal tangential components are allowed to rotate within the tangential plane perpendicular to the radial direction, with a rotation angle of θ. The initial tangential orientations of each sensor are specified in the ROT = 0° sensor array configuration. To optimize the tangential orientations, the initial tangential orientations are rotated over a predefined angular step (e.g., θ∈{0°,30°,…,330°}).

For each candidate rotation angle $\theta_{k}^{\left(c\right)}$, where c indexes the discrete angle candidates in the predefined search grid (e.g., c=1,2,3,…,12 for 30° steps), the tangential orientations of the k-th sensor are set accordingly, while the orientations of all the other sensors remain unchanged. The updated sensor configuration is denoted by $\Theta^{\left(k,c\right)}$, and the global metric is computed as

(5) $$R_{12}\left(\Theta^{\left(k,c\right)}\right)=\frac{L_{1}^{T}L_{2}}{\left\|L_{1}\right\|\left\|L_{2}\right\|}$$

The optimal direction is then selected as

(6) $$\theta_{k}^{opt}=arg\;\underset{\theta_{k}^{\left(c\right)}}{\min}{\;R}_{12}\left(\Theta^{\left(k,c\right)}\right)$$

The optimal angle $\theta_{k}^{opt}$ is assigned to sensor k, and the process is repeated for all sensors k=1,2,…,N. In each iteration, the direction of only one sensor is adjusted, but the optimization objective always reflects a global performance metric for the entire array.

## 3. Results

In this study, five experiments were designed to investigate the mechanisms influencing $\left\|L_{1}\right\|$ and $R_{12}$ and validate the proposed array optimization strategy. Experiments 1 to 4 systematically examined the effects of tangential component rotation, sensor–source alignment, source depth, and head model types, respectively. Based on these findings, Experiment 5 designed a new triaxial OPM-MEG sensor array using the RMAO method, and its effectiveness was evaluated using the $\left\|L_{1}\right\|$, $R_{12}$ and DLE.

### 3.1. Experiment 1: Effects of Tangential Orientations on $\left\|L_{1}\right\|$ *and $R_{12}$*

**Figure 3:**
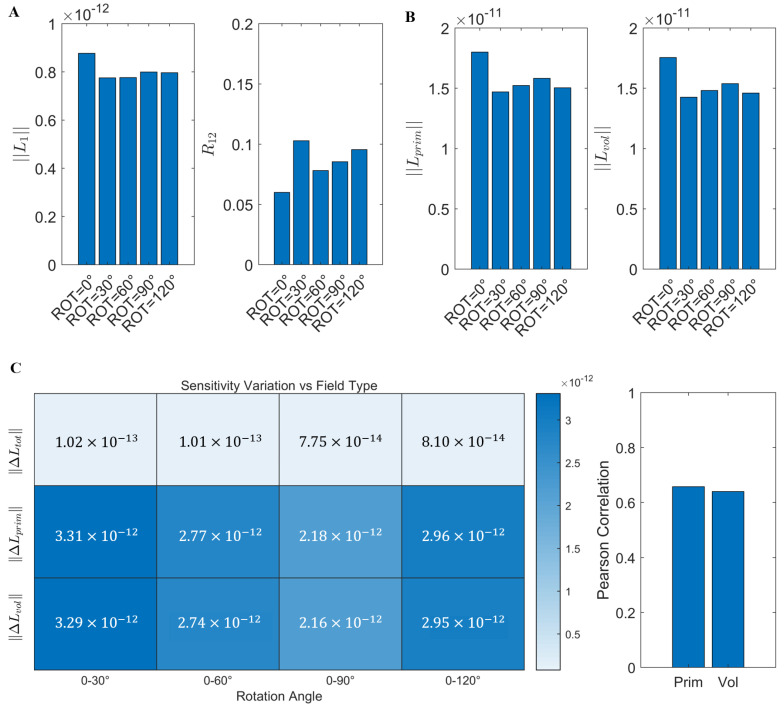
Effects of tangential component rotation on array sensitivity and lead field correlation coefficient: (**A**) Average $\left\|L_{1}\right\|$ and $R_{12}$ values for sensor arrays at five different tangential rotation angles. (**B**) Average $\left\|L_{prim}\right\|$ and $\left\|L_{vol}\right\|$ values under different rotation conditions. (**C**) Pearson correlation coefficients between the sensitivity variations in $\left\|\Delta L_{tot}\right\|$, $\left\|{\Delta L}_{prim}\right\|$, and $\left\|{\Delta L}_{vol}\right\|$.

Figure 3 shows that $\left\|L_{1}\right\|$ varied with the rotation angle, with the most significant difference observed between ROT = 0° and ROT = 30°, where the mean value of $\left\|L_{1}\right\|$ decreased by approximately 11.62%. Meanwhile, the mean value of $R_{12}$ increased from 0.0609 to 0.1020, representing an increase of about 67.50%, indicating that the rotation of the tangential component orientations significantly altered the lead field correlation between internal sources of interest and external interference sources (Figure 3A). At the level of magnetic field components, $\left\|L_{tot}\right\|$, $\left\|L_{prim}\right\|$ and $\left\|L_{vol}\right\|$ exhibited consistent trends across all the rotation angles (Figure 3B). Pearson correlation coefficients further indicated high spatial consistency in their variations ($\left\|\Delta L_{tot}\right\|$–$\left\|{\Delta L}_{prim}\right\|$: r = 0.67; $\left\|\Delta L_{tot}\right\|$–$\left\|{\Delta L}_{vol}\right\|$: r = 0.64), as shown in Figure 3C. These results suggest that although $\left\|L_{prim}\right\|$ and $\left\|L_{vol}\right\|$ originate from distinct physiological mechanisms, they still exhibit a unified response pattern to tangential component rotation. In summary, although the overall magnitude of $\left\|L_{1}\right\|$ showed limited variation across rotation angles, $R_{12}$ was more sensitive to directional perturbations, suggesting that even slight changes in measurement orientation could significantly affect the ability of the system to suppress external interference.

### 3.2. Experiment 2: Effect of Sensor–Source Alignment on $\left\|L_{1}\right\|$ *and $R_{12}$*

**Figure 4:**
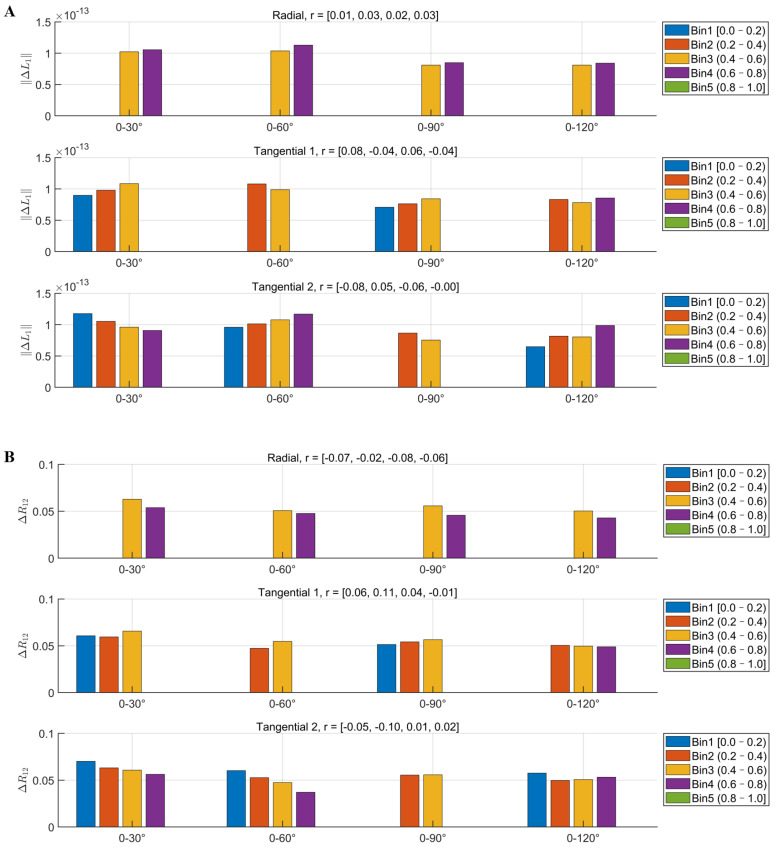
Effect of sensor–source alignment on array sensitivity and lead field correlation coefficient: (**A**) The relationship between different alignment bins (Bin1–Bin5) and the mean $\left\|\Delta L_{1}\right\|$. (**B**) The relationship between different alignment bins (Bin1–Bin5) and the mean $\Delta R_{12}$. The Pearson correlation coefficients r correspond, in order, to the four angles: 0–30°, 0–60°, 0–90°, and 0–120°.

Figure 4 showed that the radial component was primarily distributed in Bin3 and Bin4, while the tangential components (including tangential1 and tangential2) were mainly distributed across Bin1 to Bin4. Overall, neither the $\left\|\Delta L_{1}\right\|$ (Figure 4A) nor the $\Delta R_{12}$ (Figure 4B) exhibited a consistent increasing or decreasing trend across any directional component, and all the Pearson correlation coefficients were below 0.1 (with a maximum of r = 0.08). These findings suggest that compared with the rotation of tangential component orientations, the sensor–source alignment played a relatively minor role in modulating system performance, including the stability of array sensitivity and lead field structure.

### 3.3. Experiment 3: Effect of Source Depth on $\left\|L_{1}\right\|$ *and $R_{12}$*

**Figure 5:**
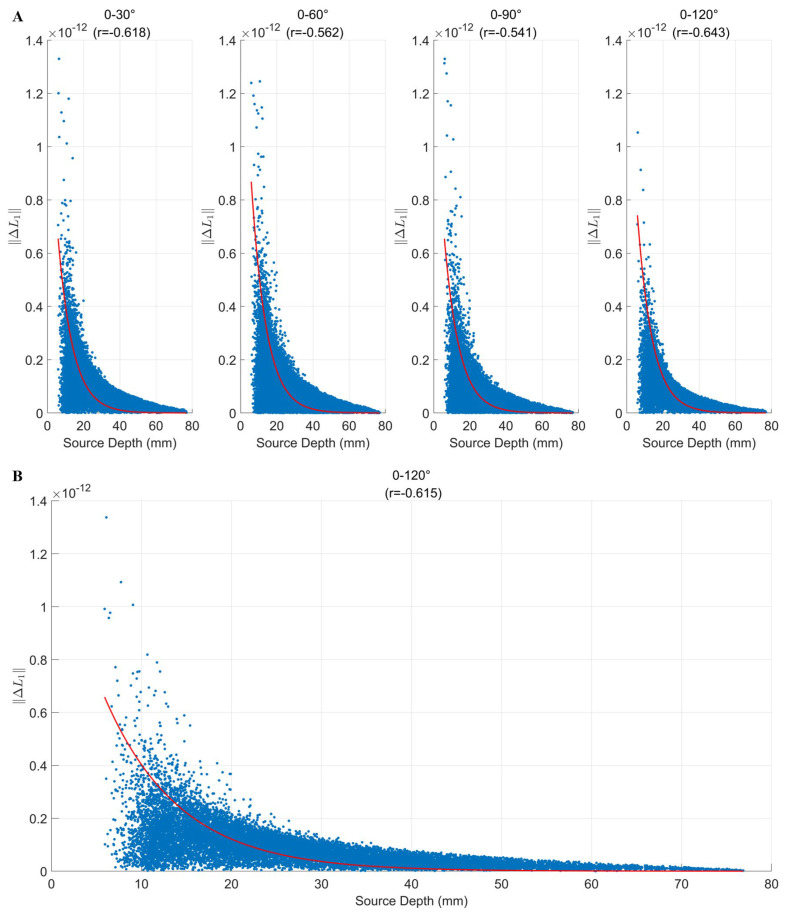
Effect of source depth on array sensitivity: (**A**) Scatter plots of source depth versus array sensitivity difference ($\left\|\Delta L_{1}\right\|$) under different tangential rotation angles. Each panel includes a regression curve (in red) and the corresponding Pearson correlation coefficient. (**B**) Relationship between the mean $\left\|\Delta L_{1}\right\|$ across all the rotation angles and source depths.

As shown in Figure 5, $\left\|\Delta L_{1}\right\|$ exhibited a significant negative correlation with source depth. Superficial sources (0–20 mm) showed the largest sensitivity differences across all the rotation angles, while deeper sources exhibited smaller variations. The correlation coefficients across the four angles ranged from r = −0.541 to −0.643, indicating a consistent trend that superficial sources are more sensitive to directional rotation (Figure 5A). When the $\left\|\Delta L_{1}\right\|$ values were averaged across all the rotation angles, a clear exponential decay trend with respect to source depth was still observed (Figure 5B), further validating the robustness of this effect.

**Figure 6:**
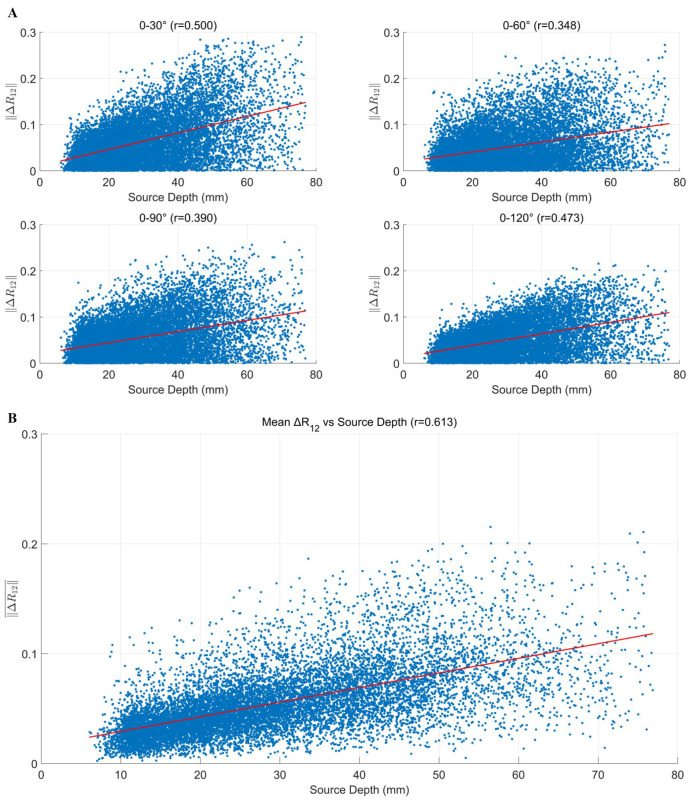
Effect of source depth on lead field correlation coefficient: (**A**) Scatter plots of source depth versus lead field correlation coefficient difference ($\Delta R_{12}$) under different tangential rotation angles. Each panel includes a regression curve (in red) and the corresponding Pearson correlation coefficient. (**B**) Relationship between the mean $\Delta R_{12}$ across all the rotation angles and source depths.

In contrast, $\Delta R_{12}$ showed a positive correlation with source depth (Figure 6A), suggesting that deeper sources exhibit more pronounced disturbances in lead field correlation. The correlation coefficients ranged from r = 0.348 to 0.500. This trend was further confirmed by the joint regression analysis (Figure 6B), where the correlation coefficient reached r = 0.613, indicating that greater source depth leads to higher instability of the lead field structure under varying measurement directions.

### 3.4. Experiment 4: Effect of Head Model Type on $\left\|L_{1}\right\|$ *and $R_{12}$*

**Figure 7:**
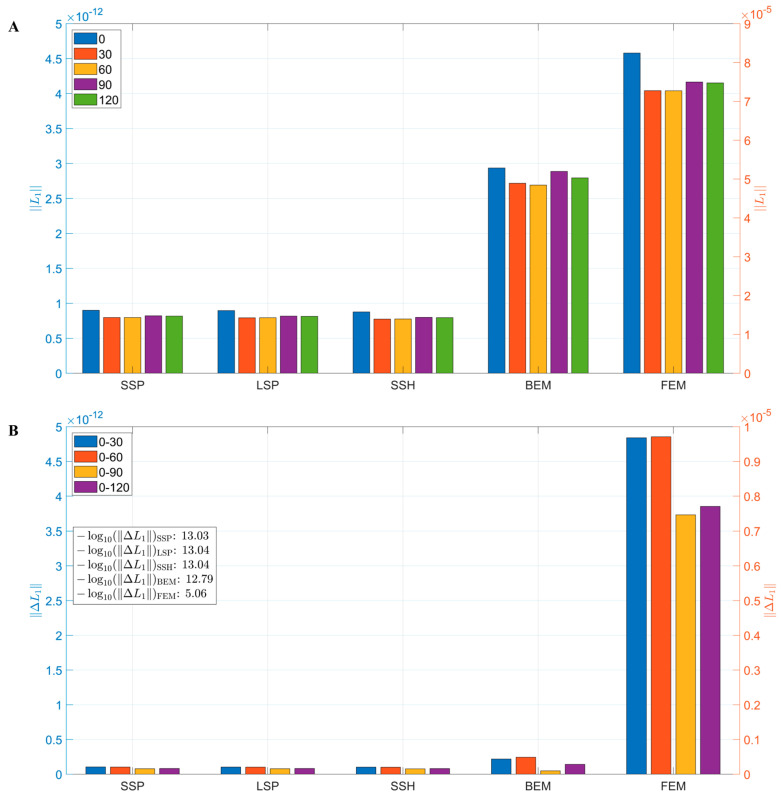
Effect of head model on sensitivity variation: (**A**) Mean $\left\|L_{1}\right\|$ values for each model across five rotation angles. The left vertical axis corresponds to the first four models (SSP, LSP, SSH, and BEM), while the right vertical axis corresponds to the FEM model due to its substantially larger magnitude. (**B**) The array sensitivity differences ($\left\|\Delta L_{1}\right\|$) between different rotation angles for each model are presented in ${-log}_{10}$ scale in the subfigure.

Figure 7A illustrates the variations in the average $\left\|L_{1}\right\|$ across different head models. The results showed that the simple models (SSP, LSP, and SSH) exhibited relatively low overall sensitivity across all the rotation angles, with minor differences between the angles. In contrast, the FEM and BEM models significantly increased overall sensitivity, particularly the FEM model, but also demonstrated a greater decline in sensitivity after rotation, indicating a higher dependence on directional precision. The difference map in Figure 7B further confirmed this trend: the FEM model exhibited a sensitivity difference between 0° and 30° (${-log}_{10}\approx 5.06$), substantially higher than the other models (${-log}_{10}\approx 13$), indicating its greater susceptibility to directional perturbations.

**Figure 8:**
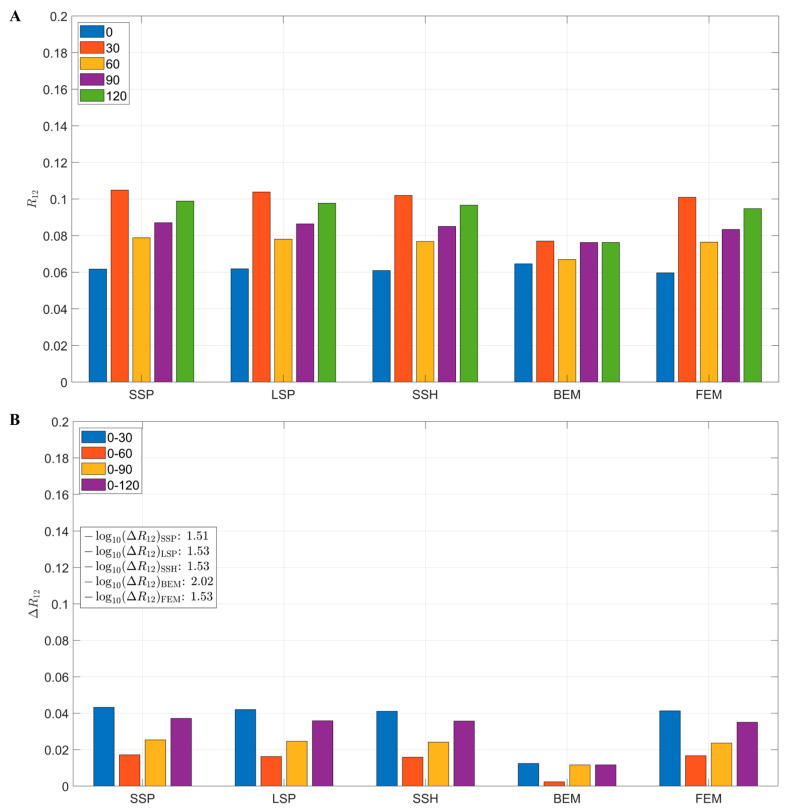
Effect of head model on lead field correlation coefficient: (**A**) Mean $R_{12}$ values for each model across five rotation angles. (**B**) The correlation coefficient differences ($\Delta R_{12}$) between different rotation angles for each model are presented in ${-log}_{10}$ scale in the subfigure.

Figure 8A illustrates the variation trends of the average $R_{12}$ across different head models. The results showed that all the models exhibited some degree of change in $R_{12}$ as the rotation angle varied. Among them, SSP, LSP, SSH, and FEM displayed relatively consistent patterns, whereas the BEM model showed comparatively smaller changes. The correlation coefficient difference ($\Delta R_{12}$) diagram in Figure 8B further indicated that the BEM model maintained the most stable lead-field structure under different rotation conditions (${-log}_{10}\approx 2.02$), while the FEM and other models exhibited greater differences (${-log}_{10}\approx 1.5$). These findings suggest that although the sensitivity stability of the BEM model was slightly lower than that of the SSP, LSP, and SSH models, it demonstrated superior robustness in terms of lead field structural stability.

### 3.5. Experiment 5: Evaluation of the Effectiveness of Array Optimization

To evaluate the effectiveness of sensor array optimization, we constructed simulated OPM-MEG data and tested performance using both sensor-level and source-level metrics. In the simulation dataset, two dipolar sources were activated simultaneously. The intracerebral source of interest was randomly selected from a distributed source model. The source signal was Gaussian-distributed data sampled at 1000 Hz, with a fixed amplitude of $Q_{1}=10\;nAm$. The lead field matrix $L_{1}$ was computed using the SSH model. The extracerebral source of interference was randomly selected from a predefined noise model, and its signals were also generated from Gaussian random data. The amplitude of the external interference sources was set to $Q_{2}=\alpha Q_{1}$, where α was a scaling factor controlling the interference level. The corresponding lead field matrix $L_{2}$ was computed based on the Biot–Savart law [36]. Sensor noise was modeled as Gaussian random noise with a standard deviation of 30 fT, and was assumed to be independent across sensors. The OPM-MEG simulated data were generated by summing the internal brain source signal, external interference, and sensor noise. The data duration was 1000 ms, with the first 200 ms used as the baseline period. In each simulation iteration, a different pair of internal interest and external interference sources was randomly selected. The interference coefficient α was varied as α=0,1,3,5,7,9 to evaluate the interference rejection performance of the system. Beamforming was performed using the linearly constrained minimum variance (LCMV) method [37] implemented in the FieldTrip toolbox (Version 20220202) [33]. The data covariance matrix was computed over the entire time window, and the regularization parameter was determined based on the estimated noise level of the data [38,39].

**Figure 9:**
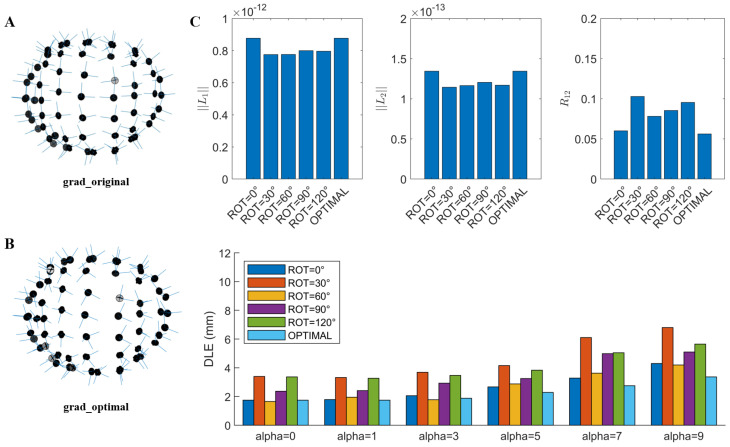
Evaluation of the effectiveness of optimization of triaxial sensor arrays based on RMAO: (**A**) The original triaxial array at ROT = 0°. (**B**) The optimization triaxial array. (**C**) Evaluation of sensor- and source-level metrics.

Based on the above findings, we selected the triaxial sensor array with the best performance at ROT = 0° as the original array (Figure 9A). Subsequently, a new triaxial sensor array was designed using the RMAO method (Figure 9B). To validate the effectiveness of this method, we computed both sensor-level and source-level metrics of the optimized array and compared them with those of arrays at different rotation angles (Figure 9C). The results showed that the optimized triaxial array improved the mean value of $\left\|L_{1}\right\|$ metric by 0.2 × 10^−13^ T, reduced the mean value of $R_{12}$ by 0.014, and decreased the mean value of DLE by 0.05–1.27 mm. It is worth noting that the estimation error of beamformers is a nonlinear function of $R_{12}$. Therefore, even a seemingly minor reduction in $R_{12}$ can lead to substantial improvements in beamformer performance, especially when the external interference is strong [15].

## 4. Discussion

Triaxial OPM-MEG, with its highly flexible sensor configurations, has opened up new design possibilities for building high-performance and personalized neural signal acquisition systems. The existing research has primarily focused on the impact of sensor number, spatial arrangement, and physical structure on system performance, covering aspects such as neural signal acquisition efficiency [40], spatial sampling density [41], source localization capability [42,43], and physical structural constraints [26,44]. In contrast, studies on the configuration strategies for tangential components remain limited. To address this issue, this study systematically investigated the role of the orientation of tangential components in enhancing sensitivity and suppressing interference, and proposed the RMAO strategy, which provides a feasible solution at both the structural and mechanistic levels. The results have demonstrated that optimizing the tangential components configuration not only improves key performance metrics ($\left\|L_{1}\right\|$, $R_{12}$, and DLE), but also offers a theoretical foundation and technical support for the engineering design of future efficient and customizable OPM-MEG systems.

First, Experiment 1 demonstrated that the rotation of tangential components significantly altered key performance metrics, particularly the sensitive response of the lead field correlation coefficient $R_{12}$. With only a slight rotation from 0° to 30°, $R_{12}$ increased by more than 67%, while $\left\|L_{1}\right\|$ simultaneously decreased by 11%. This nonlinear response suggests that array performance is highly sensitive to directional perturbations, especially in terms of lead field structure. In addition, although the magnetic field is jointly determined by $L_{prim}$ and $L_{vol}$, these components exhibited consistent trends under direction rotation, indicating a coordinated and tightly coupled response of different magnetic field components to directional changes. This further highlights the significance of optimizing direction configuration in controlling the overall magnetic field structure.

Second, Experiments 2 and 3 further investigated the modulatory mechanisms by which the spatial properties of sources influence the effects of directional perturbations. The results of Experiment 2 showed that the sensor–source alignment had a minimal effect on sensitivity, with correlations approaching zero. This finding indirectly suggests that compared to the factor of “directional alignment with the source,” the overall spatial distribution pattern of tangential components may exert a greater impact on signal detection and interference coupling. Experiment 3 emphasized the role of source depth as a key modulating variable affecting the influence of directional changes. Superficial sources exhibited high sensitivity of ***L*_1_** to directional variation, with a significant negative correlation, while deep sources demonstrated greater instability in *R*_12_, indicating a trade-off in optimization goals across different depths. For superficial sources, priority should be given to preserving sensitivity, whereas for deep sources, maintaining robustness of the lead field structure becomes essential.

Third, Experiment 4 compared five types of head models and revealed an inherent trade-off between modeling fidelity and robustness to direction perturbation. The results showed that simplified models (SSP, LSP, and SSH), due to their coarse approximation of volume current distribution [45], exhibited good rotational robustness but relatively low sensitivity. The FEM model demonstrated the highest sensitivity magnitude but also the greatest fluctuation under rotation due to its high sensitivity to anatomical detail [46]. The BEM model achieved a good balance between the two. These findings suggest that while high-fidelity models can improve performance, they may also amplify directional error sensitivity. Thus, in practical applications, a balance must be struck between precision and robustness. For scenarios requiring wide-range directional control, models with greater structural stability, such as BEM, may offer more practical advantages.

Fourth, based on the aforementioned mechanistic findings, Experiment 5 implemented the RMAO strategy and systematically evaluated the performance of the optimized array. The results demonstrated that RMAO substantially improved sensitivity to cortical sources and effectively suppressed external interference coupling, thereby enhancing source localization accuracy. In particular, when the system was under strong interference conditions (α=9), the optimized array reduced the average DLE by 1.27 mm. This has important implications for clinical scenarios requiring high-precision source localization, such as epileptogenic focus resection, preoperative functional area mapping, and deep brain stimulation target planning. In these applications, even a 1–2 mm improvement in MEG localization accuracy can significantly reduce surgical boundary uncertainty, increase the likelihood of complete lesion removal, and minimize the risk of damage to surrounding critical cortical regions. Moreover, although the numerical reduction in $R_{12}$ was relatively modest, its nonlinear influence on beamformer performance indicated that even minor optimizations could yield significant system-level benefits, particularly under high-interference conditions. This finding serves to both validate the effectiveness of the RMAO method and reinforce the theoretical rationale for adopting $R_{12}$ as a key optimization objective.

Finally, to evaluate the impact of rotation step size on optimization results, we conducted additional rotation optimization tests using smaller step sizes (20° and 10°), as presented in the Supplementary Material S1. The results show that under strong intensity external interference conditions (α=9), smaller rotation steps can indeed lead to better performance, such as further reductions in $R_{12}$ and lower average DLE values. Although the performance gains are notable, the improvement is not linearly proportional to the decrease in step size. It is important to note that reducing the step size significantly increases the dimensionality of the search space, resulting in a substantial rise in computational cost. For example, with a 10° step, the number of candidate directions for a single sensor expands to 36, which is three times that of the 30° step, leading to an approximately threefold increase in computation time. Therefore, we recommend selecting an appropriate rotation step size in practical applications based on the specific requirements for performance and available computational resources, in order to achieve a reasonable balance between optimization effectiveness and computational efficiency.

Moreover, recent studies have shown that alternately arranged biaxial sensor arrays can also achieve good three-dimensional sensitivity coverage [47]. Although this study focused on triaxial sensor arrays, the proposed method is equally applicable to the further optimization of biaxial arrays. In the biaxial configuration, each sensor measures one radial component and one tangential component, and the optimization process only needs to adjust one tangential measurement component within the tangential plane perpendicular to the radial component. We implemented this adapted version in Supplementary Material S2 and evaluated its performance under the same simulation conditions. The results showed that the optimized biaxial array outperformed the non-optimized array in reducing $R_{12}$ and decreasing DLE, and exhibited better robustness under strong interference conditions (α=9). Although the performance improvement was slightly smaller than that of the triaxial array, the biaxial configuration still offers advantages in reducing hardware complexity and suppressing crosstalk, and therefore has practical value in specific applications.

## 5. Limitations

In the modeling framework of this study, we assumed independence between sensors to simplify the analysis and highlight the effects of lead field structure and sensor orientation. However, in real OPM-MEG systems, particularly in densely packed triaxial sensor arrays, magnetic crosstalk and mutual interference may occur between sensors, thereby introducing correlations between channels [48]. Such crosstalk can alter the effective gain and sensitive axis orientation of sensors, and have a significant impact on source discriminability under high SNR conditions. Its magnitude is closely related to the physical layout of the sensors and is most severe when the magnetic field direction of a neighboring sensor is collinear with the sensitive axis of the target sensor and the inter-sensor spacing is small [49]. Future research will consider incorporating crosstalk effects into the modeling process to enhance the robustness and applicability of the optimization framework under real measurement conditions.

In addition, the experimental validation stage of this study is constrained by the current laboratory conditions and is still in the simulation exploration stage, without yet conducting physical experimental validation in a real OPM-MEG system. We anticipate that, in practical applications, multiple challenges may arise, including insufficient sensor calibration accuracy, involuntary subject movement, and variations in external interference environments. To address these challenges, we plan to employ accurate three-dimensional positioning and online self-calibration algorithms [50], real-time motion tracking and compensation mechanisms [51], and adaptive interference suppression methods incorporating adjustable reference sensor layouts [52]. Furthermore, in the simulation stage of this study, we assumed that sensor noise follows a Gaussian distribution and that external interference sources are represented by an idealized model. Such simplifications may not fully capture the complexity of real measurement environments. Future work will incorporate empirical noise and external interference data from actual OPM-MEG systems to further validate the proposed optimization method, thereby enhancing the robustness and practical applicability of the study’s conclusions. As experimental conditions mature, we will continue to carry out relevant experimental research and evaluate the proposed method under multiple tasks and multiple subjects, in order to verify its effectiveness and robustness in real-world application scenarios.

## 6. Conclusions

This study systematically evaluated the impact of tangential components configurations in OPM sensor arrays on $\left\|L_{1}\right\|$, $R_{12}$, and DLE, and proposed an optimization method based on RMAO. The experimental results showed that the optimized triaxial sensor array increased the mean value of $\left\|L_{1}\right\|$ metric by 0.2 × 10^−13^ T, reduced the mean value of $R_{12}$ by 0.014, and decreased the mean value of DLE by 0.05–1.27 mm. This work provides theoretical support and methodological guidance for the structural optimization and system design of OPM-MEG sensor arrays.

## Data Availability

The data, aside from the data published in this manuscript, are not publicly available due to privacy restrictions.

## Supplementary Materials

The following supporting information can be downloaded at: https://www.mdpi.com/article/10.3390/bioengineering12090903/s1, Supplementary Material S1: Simulation Tests of Directional Optimization Under Different Rotation Steps. Supplementary Material S2: Adaptation and Performance Evaluation of the RMAO Method in Biaxial OPM-MEG Arrays.
