# Supplementary material for: Optimization and Analysis of Tangential Component Orientations in OPM-MEG Sensor Array
Source: Bioengineering (Basel). 2025 Aug 22;12(9):903. doi: 10.3390/bioengineering12090903 (PMC12467417; doi:10.3390/bioengineering12090903)
Supplement: Supplementary file 1 [file bioengineering-12-00903-s001.zip › bioengineering-3766963-supplementary.pdf]

Supplementary Material

Supplementary Material S1: Simulation Tests of Directional Optimization Under Different Rotation Steps

To evaluate the impact of rotation step size on array optimization performance, we extended the original simulation framework based on the RMAO method by adding directional optimization tests with smaller step sizes (20°and 10°). The simulations were conducted using a consistent triaxial OPM sensor array structure, with three tested step sizes: 30°, 20°, and 10°. All tests were performed under the same high-intensity external interference condition ($\alpha = 9$), in order to highlight the optimization differences caused by varying step sizes. The evaluation metrics included R_{12}, DLE, and computation time.

The results showed that as the rotation step size decreased from 30°to 10°, the optimized arrays exhibited consistent improvements across performance metrics (shown as in Table S1). Specifically, compared to the 30° step size, smaller step sizes yielded lower average R_{12} values and further reductions in average DLE, indicating that both the system's interference suppression capability and source localization accuracy were improved. However, the increase in computational cost caused by smaller step sizes was also substantial. The number of candidate directions per sensor increased from 12 to 36 under the 10°step, which is three times that of the 30°step; accordingly, the total search space and computation time also nearly tripled. Due to the exponential growth of the search space, the practical feasibility of very small step sizes is limited.

In summary, smaller rotation step sizes can indeed improve the performance of directional optimization, particularly in complex interference environments. However, they also significantly increase the consumption of computational resources. Therefore, in practical applications, the rotation step size should be selected based on the specific performance requirements and available computational resources, in order to achieve an optimal balance between optimization effectiveness and computational efficiency.

Table S1. Performance Comparison Under Different Rotation Step Sizes

Step Size	Candidate Directions per Sensor	Mean R_{12}	Mean DLE	Computation Time
**30°**	12	0.0562	3.36 mm	40251.39 s
**20°**	18	0.0524	3.18 mm	65434.15 s
**10°**	36	0.0487	2.94 mm	118303.50 s

Supplementary Material S2: Adaptation and Performance Evaluation of the RMAO Method in Biaxial OPM-MEG Arrays

To verify the applicability of the proposed RMAO method beyond triaxial OPM-MEG arrays, we conducted supplementary experiments in which the optimization strategy was adapted to a biaxial sensor array. The objective of this supplementary experiment was to evaluate the optimization effectiveness of the RMAO method in biaxial arrays and to quantify its performance improvements relative to the unoptimized configuration.

The simulation experiments were kept consistent with those of the triaxial array, including the same head model, source space, and external interference conditions, to ensure the comparability of results. In the adapted optimization framework, the search space for each biaxial sensor was restricted to the tangential plane perpendicular to its radial component, with only the single tangential component direction being optimized. The optimization objective was kept the same as in the triaxial array, i.e., to always ensure the minimization of the global array R_{12}. The performance evaluation metrics included array sensitivity, average R_{12}, and average DLE.

As shown in Figure S1, applying the adapted RMAO method to the biaxial array led to significant performance improvements compared to the unoptimized biaxial array. Specifically, the optimized average $\|\boldsymbol{L_1}\|$ value increased by 0.18×10^{-13} T, the average R_{12} value decreased by 0.010, and the average DLE was further reduced, while exhibiting better robustness under high-interference conditions ($\alpha = 9$). The optimization trends were consistent with those of the triaxial array; however, due to the reduced degrees of freedom, the absolute magnitude of performance improvement was slightly smaller. Nevertheless, the results demonstrate that biaxial arrays guided by the RMAO method still possess substantial optimization potential, achieving noticeable improvements in interference suppression capability and source localization accuracy.

In conclusion, the proposed RMAO method is not limited to triaxial OPM-MEG arrays but is also applicable to biaxial configurations. Although the absolute performance gains are slightly lower due to the reduced optimization freedom, the lower hardware complexity and reduced crosstalk risk make biaxial arrays highly valuable in certain practical MEG applications. The findings of this study extend the applicability of the RMAO framework and provide a reference for sensor array design under different hardware conditions.

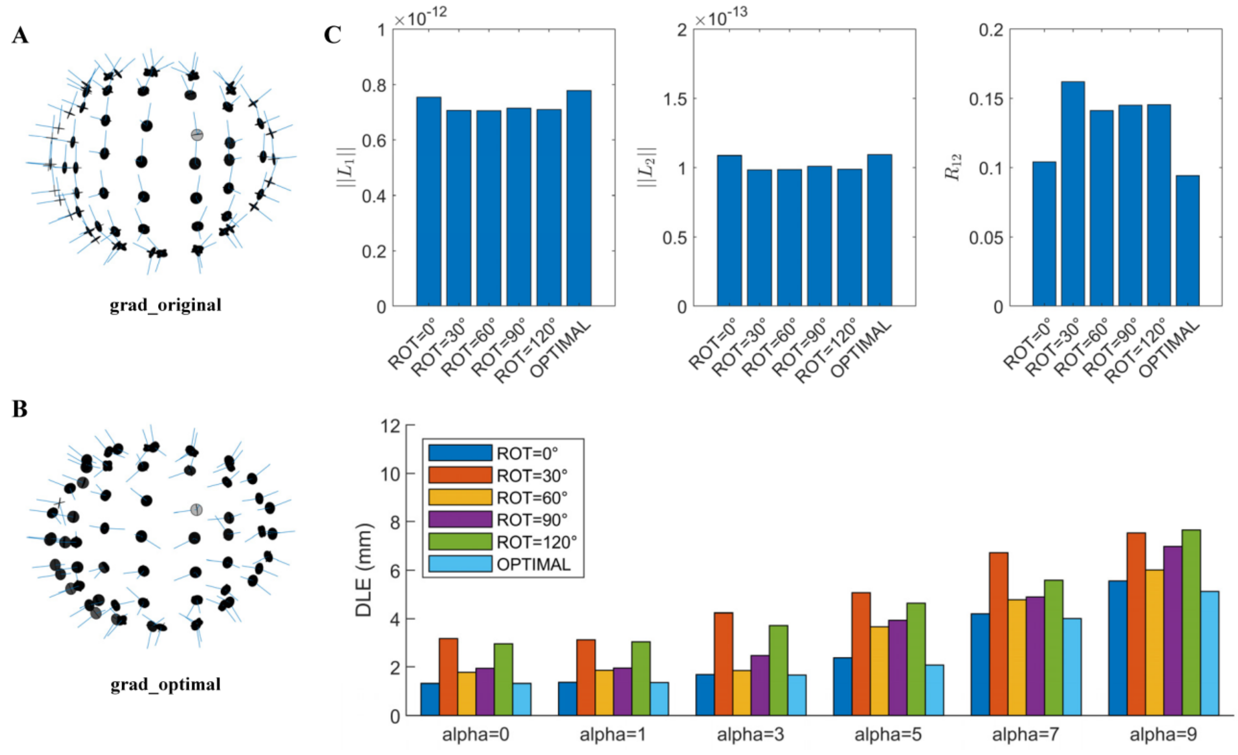

Figure S1. Evaluation of the effectiveness of optimization of biax sensor arrays based on RMAO. (A) The original biax array at ROT = 0°. (B) The optimization biax array. (C) Evaluation of sensor- and source-level metrics.
